# Efficacy of an Endoscopic Device Integrating a Sphincterotome and a Dilation Balloon Catheter for the Treatment of Choledocholithiasis (with Video)

**DOI:** 10.3390/jcm14175930

**Published:** 2025-08-22

**Authors:** Jun-Ichi Hanatani, Koh Kitagawa, Shohei Asada, Yuki Motokawa, Yui Osaki, Tomihiro Iwata, Yukihisa Fujinaga, Norihisa Nishimura, Kosuke Kaji, Shinya Sato, Tadashi Namisaki, Akira Mitoro, Hitoshi Yoshiji

**Affiliations:** 1 Department of Gastroenterology, Nara Medical University, Nara 6348522, Japan; 2 Division of Endoscopy, Nara Medical University, Nara 6348522, Japan; mitoroak@naramed-u.ac.jp

**Keywords:** choledocholithiasis, common bile duct stone, endoscopic sphincterotomy, endoscopic papillary balloon dilation, endoscopic papillary large balloon dilation

## Abstract

**Background/Objectives:** The combined techniques of endoscopic sphincterotomy followed by endoscopic papillary balloon dilation (ESBD) and endoscopic sphincterotomy followed by endoscopic papillary large balloon dilation (ESLBD) have gained popularity for the endoscopic treatment of choledocholithiasis. However, the conventional approach to these procedures requires two separate devices, a sphincterotome and a balloon catheter, which can complicate and prolong the procedure. We herein evaluated a novel integrated device combining a sphincterotome and balloon catheter developed to improve the efficiency of ESBD and ESLBD. **Methods:** This retrospective study compared the clinical outcomes of patients with choledocholithiasis who were treated using conventional sphincterotome and balloon catheters (*n* = 106) and those who were treated using the integrated device (*n* = 54). **Results:** Overall complete stone removal rates (99.1% vs. 100%) and adverse event incidence (12.3% vs. 13.0%) were comparable between the two groups. However, the integrated device significantly reduced total procedure time (40 vs. 27 min, *p* = 0.01), use of mechanical lithotripter (50.0% vs. 22.2%, *p* < 0.01), total number of procedures required. **Conclusions:** Complete stone removal rates and safety were comparable between the two groups. However, the novel integrated device may enhance the efficiency of common bile duct stone removal through ESBD and ESLBD because it eliminates the need to prepare and exchange separate devices.

## 1. Introduction

Choledocholithiasis has been identified as a major cause of acute cholangitis and pancreatitis, both of which are serious abdominal issues [1]. With the rapid aging of the global population [2], the number of elderly patients with choledocholithiasis is expected to increase. Therefore, safe and efficient stone removal in the shortest possible time has become increasingly important. Endoscopic retrograde cholangiopancreatography (ERCP) is a widely employed technique for treating common bile duct (CBD) stones. Ampullary intervention is a critical step in this procedure, and various techniques have been developed to optimize outcomes. Endoscopic sphincterotomy (EST), first described by Kawai et al. in 1974, has become the standard ampullary intervention for CBD stone removal [3]. However, EST requires considerable technical expertise and carries risks, including bleeding, perforation, and Oddi sphincter dysfunction [4,5,6,7]. Endoscopic papillary balloon dilation (EPBD), introduced by Staritz et al. in 1983 [8], demands less technical expertise and is associated with a lower risk of bleeding and perforation, while preserving Oddi sphincter function. However, EPBD has been reported to carry a higher risk of post-ERCP pancreatitis (PEP) [9]. Furthermore, endoscopic papillary large balloon dilation (EPLBD) has been shown to be effective for removing large stones, as initially reported by Ersoz et al. in 2003 [10].

In recent years, combined techniques such as ESBD (EST followed by EPBD) and ESLBD (EST followed by EPLBD) have gained popularity. Several studies have demonstrated the effectiveness of these approaches. A retrospective study by Ishii et al. showed that ESBD significantly reduced procedure time, overall adverse events, and bleeding, while achieving a higher single-session complete stone removal rate compared to EST alone [11]. Similarly, randomized controlled trials and retrospective studies have reported that ESLBD achieves significantly higher single-session complete large-stone removal rates and shorter procedure times compared to EST alone [12,13,14]. Despite their effectiveness, conventional ESBD and ESLBD require two separate devices: a sphincterotome and a balloon catheter. This can make the procedure more cumbersome and time-consuming. Device exchange may pose a significant burden, especially for trainees. To address these limitations, a novel integrated device was developed, combining a sphincterotome and a balloon catheter into a single tool, to facilitate more efficient ESBD and ESLBD procedures [15]. Currently, this integrated device has been widely available and utilized in various regions, including Europe, North America, and Asia.

The objective of this study was to examine the efficacy of the novel integrated device for the treatment of choledocholithiasis.

## 2. Materials and Methods

### 2.1. Study Population

**Figure 1:**
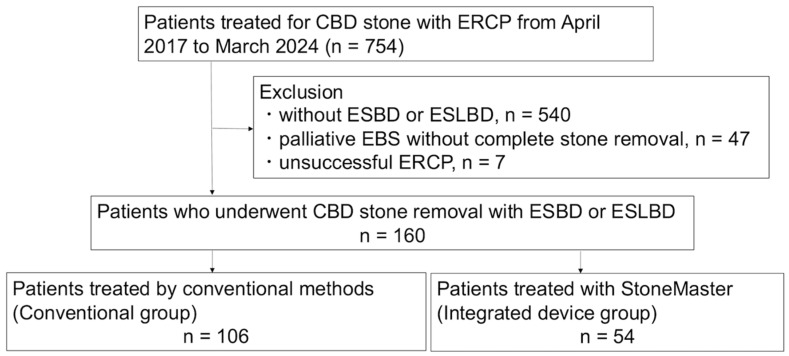
Schematic illustration of the study design and patient selection criteria. CBD, common bile duct; ERCP, endoscopic retrograde cholangiopancreatography; ESBD, endoscopic papillary balloon dilation with endoscopic sphincterotomy; ESLBD, endoscopic papillary large balloon dilation with endoscopic sphincterotomy.

This single-center retrospective study was approved by the Nara Medical University Ethics Committee (#3980). Figure 1 illustrates the patient flow chart. Between April 2017 and March 2024, a total of 754 patients underwent ERCP for CBD stone removal at our institution. The diagnosis of CBD stone was established prior to ERCP using abdominal ultrasonography, computed tomography, magnetic resonance cholangiopancreatography, or endoscopic ultrasonography. We excluded 540 patients who did not undergo ESBD or ESLBD, 47 patients who received palliative endoscopic biliary stenting without stone removal, and 7 patients with unsuccessful ERCP. The remaining 160 patients who underwent CBD stone removal using ESBD or ESLBD were eligible for this study.

ESBD was defined as EST followed by EPBD using a dilated balloon size of <12 mm (Video S1). ESLBD was defined as EST followed by EPLBD using a dilated balloon size of ≥12 mm (Video S2). The 160 patients were divided into two groups based on the device used for ampullary intervention: the conventional group (*n* = 106), which used a conventional sphincterotome and a balloon catheter, and the integrated device group (*n* = 54), which used an endoscopic device integrating a sphincterotome and a dilation balloon catheter. This study adhered to the Strengthening the Reporting of Observational Studies in Epidemiology (STROBE) Statement. Written informed consent was obtained from all patients before ERCP. Given the study’s retrospective nature, an opt-out approach was employed instead of requiring written informed consent for participation.

### 2.2. Endpoints

The primary endpoint was the rate of complete stone removal. The secondary endpoints were procedure time, length of hospital stay, and incidence of adverse events.

### 2.3. ERCP

During ERCP, patients were primarily positioned in the prone position, although some patients underwent the procedure in the left lateral or supine position. Anesthesia or sedation was administered with midazolam and buprenorphine hydrochloride or haloperidol and dexmedetomidine hydrochloride, as deemed appropriate. Vital signs were continuously monitored using electrocardiography and oxygen saturation assessments throughout the procedure. Oxygen supplementation via nasal cannula was provided when necessary. A side-viewing endoscope (JF-260V, TJF-260V, TJF-Q290V; Olympus Medical, Tokyo, Japan) was used. Selective biliary cannulation was performed using a standard ERCP catheter (MTW ERCP catheter; MTW Endoskopie, Wesel, Germany) with a wire-guided technique in conjunction with contrast medium injection.

The diagnosis of acute cholangitis was based on the Tokyo Guidelines 2018 [16]. Adverse events were classified and graded based on the severity grading system outlined in the Lexicon of the American Society for Gastrointestinal Endoscopy [17]. The total procedure time was defined as the time from when the duodenoscope reached the papilla of Vater to when the duodenoscope was removed.

### 2.4. ESBD and ESLBD

**Figure 2:**
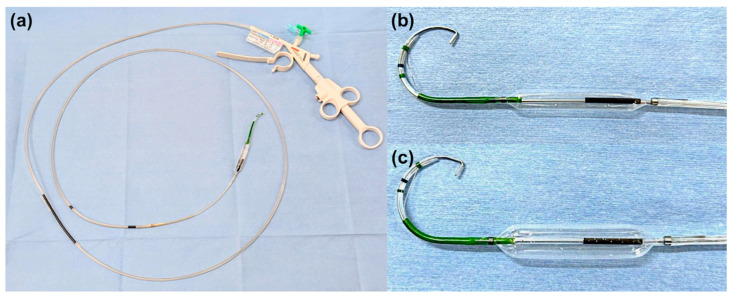
(**a**) An endoscopic device integrating a sphincterotome and a balloon (StoneMaster V; Olympus Corp., Tokyo, Japan); (**b**,**c**) Two types of balloon sizes can be used for ESBD (endoscopic papillary balloon dilation with endoscopic sphincterotomy) and ESLBD (endoscopic papillary large balloon dilation with endoscopic sphincterotomy).

**Figure 3:**
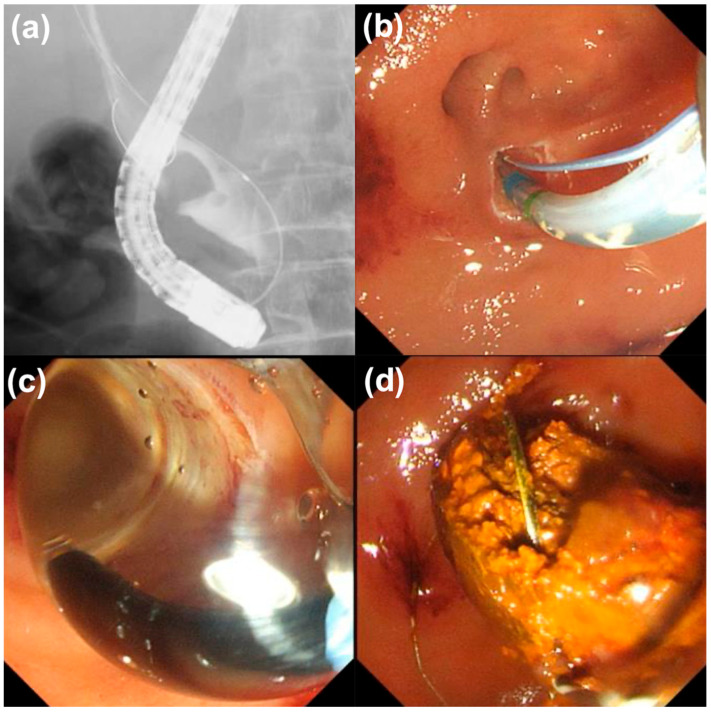
Intraoperative photographs showing common bile duct (CBD) stone removal via endoscopic papillary large balloon dilation with endoscopic sphincterotomy using a large-type integrated device. (**a**) Fluoroscopic image: cholangiogram shows a 15 mm CBD stone; (**b**,**c**) Endoscopic images: First, endoscopic sphincterotomy (small incision) was performed, followed promptly by endoscopic papillary large balloon dilation. (**d**) The CBD stone was removed using a mechanical lithotripter without crushing the stone.

In the conventional group, EST was performed using a sphincterotome (CleverCut 3V; Olympus Medical, Tokyo, Japan), followed by papillary balloon dilation with a balloon catheter (Hurricane ^TM^ RX, CRE wire-guided balloon dilator; Boston Scientific, Natick, MA, USA, REN; Kaneka Medix Corp., Osaka, Japan). In the integrated device group, EST and papillary balloon dilation were performed using an integrated device (StoneMaster V; Olympus Medical, Tokyo, Japan; Figure 2 and Figure 3) [15]. EST incision size was either small (not extending over the hooding fold) or medium (extending over the hooding fold). The choice of balloon dilation diameter and the decision to perform ESBD or ESLBD were primarily based on the stone diameter and CBD size. The papillary dilation procedure was performed by inflating the balloon under fluoroscopic guidance until the balloon waist disappeared, holding the inflation for 30 s. Stone removal was achieved using a basket catheter (Medi-Globe 8-Wire Nitinol Basket; Medi-Globe GmbH, Rohrdorf, Germany), a balloon catheter (Extraction balloon catheter; ZEON Medical Inc, Tokyo, Japan), and/or a mechanical lithotripter (XEMEX Lithotripsy Basket Catheter; ZEON Medical Inc., Tokyo, Japan). Complete stone removal was assessed using contrast cholangiography performed at the end of the ERCP to confirm stone clearance.

### 2.5. Statistical Analysis

Statistical analyses were performed using EZR version 1.41 (Saitama Medical Center, Jichi Medical University, Saitama, Japan) [18], a graphical user interface for R version 4.2.2 (The R Foundation for Statistical Computing, Vienna, Austria). Continuous variables were presented as mean ± standard deviation or median (range), as appropriate, while categorical variables were summarized as frequency (percentage). Comparisons between the two groups were made using the *t*-test or Mann–Whitney U test for continuous variables with skewed distributions, and the chi-squared test or Fisher’s exact test for categorical variables. *p*-values < 0.05 were considered indicative of statistical significance.

## 3. Results

### 3.1. Baseline Characteristics

Table 1 summarizes the baseline characteristics and demographic data of patients upon admission. The integrated device group had a significantly higher proportion of patients with periampullary diverticulum compared to the conventional group. The integrated device group had a significantly lower proportion of patients with a WHO performance status score of 0–1. There were no significant between-group differences in terms of age, sex, antiplatelet or anticoagulant therapy, cardiovascular disease, history of cerebrovascular disease, dementia, history of malignant neoplasm, Billroth I reconstruction, acute cholangitis, severity of cholangitis, gallstone-associated pancreatitis, number of stones, stone diameter, or CBD diameter. None of the patients had surgically altered anatomy, except for Billroth I reconstruction. The median follow-up period for all cases was 165.5 days.

### 3.2. Impact of the Integrated Device on CBD Stone Removal

Table 2 summarizes the endoscopic procedures. The ESBD to ESLBD ratio did not differ significantly between the groups. Both groups had high rates of successful complete stone removal 99.1% [105/106] in the conventional group and 100% [54/54] in the integrated device group, with no significant between-group difference. However, the integrated device group had a significantly higher rate of single-stage stone removal than the conventional group (conventional group: 40.6%; integrated device group: 61.1%; *p* = 0.02). The integrated device group required fewer sessions for complete stone removal and had shorter procedure times than the conventional group (conventional group: 40 min; integrated device group: 27 min; *p* = 0.01). Endoscopic mechanical lithotripsy (EML) was significantly less used in the integrated device group (conventional group: 50.0%; integrated device group: 22.2%; *p* < 0.01). Although the time required for selective biliary cannulation did not differ significantly, it was slightly shorter in the integrated device group than in the conventional group. There were no significant differences between the two groups in terms of the use of pancreatic guidewires, pancreatic stents, or precutting techniques. No patients in either group received rectal diclofenac administration prior to ERCP. In addition, the integrated device group had a shorter length of hospital stay (the conventional group, 12 days; integrated device group, 8 days; *p* = 0.02).

Table 3 also shows adverse events related to ERCP. There was no significant difference in the overall incidence of adverse events between the two groups. All patients who developed pancreatitis, cholangitis, deterioration of respiratory condition, or aspiration pneumonia in both groups improved with conservative treatment. No cases of pancreatitis occurred in the integrated device group. Bleeding occurred in six patients in the conventional group and three patients in the integrated device group. The details and management of bleeding cases are shown in Table 4. Hemostasis was achieved endoscopically in all patients without requiring angiographic intervention or surgical operation. Immediate bleeding during the ERCP session occurred in four patients in the conventional group and two patients in the integrated device group. In the conventional group, three (75.0%) patients required diluted epinephrine spray, and all four (100%) required balloon tamponade. In contrast, both patients (100%) in the integrated device group required diluted epinephrine spray. Delayed bleeding after the ERCP session occurred in two patients in the conventional group, and one patient in the integrated device group. Blood transfusion was required for one patient in the conventional group. Hemostasis was achieved with diluted epinephrine spray and balloon tamponade in one patient, and with injection of hypertonic saline epinephrine solution and hemostatic clipping in the other. The single patient in the integrated device group achieved hemostasis with balloon tamponade. There were no procedure-related deaths in either group.

### 3.3. Comparison Based on Stone Size

Table 5 compares the endoscopic procedures and adverse events between the small- and large-stone groups. Accordingly, the small-stone group comprised patients with stones measuring ≤ 10 mm in diameter, whereas the large-stone group comprised those with stones > 10 mm. The large-stone group underwent ESLBD and used mechanical lithotripsy more frequently as well as had a longer procedure time than the small-stone group. Conversely, no significant differences in the usage rates of the integrated device, complete stone removal rates, single-stage stone removal rates, total number of sessions required for complete stone removal, length of hospital stay, or incidence of adverse events were observed between the two groups.

## 4. Discussion

In this study, overall complete stone removal rates and safety were comparable between the two groups. However, the novel integrated device significantly reduced total procedure time and number of procedures required. The decreased procedure time can be attributed to the elimination of the need for separate preparation and exchange of sphincterotome and balloon catheter during ESBD and ESLBD. Moreover, the reduced technical burden on endoscopists during ampullary intervention may have facilitated smoother subsequent stone extraction, contributing to fewer procedures. In fact, despite no difference in the size or number of stones between the two groups, the use of EML was lower in the integrated device group than in the conventional group. Although the shortening of the hospitalization period was unlikely to be a direct effect of this device, it may be attributed to the reduced number of procedures required.

The debate surrounding the optimal approach for extracting bile duct stones via ampullary intervention continues, with some advocating for EST alone and others recommending a combination of EST and balloon dilation. Although utilizing two devices (sphincterotome and balloon catheter) can add complexity to the ERCP procedure, it can compensate for the respective limitations of EST and EPBD/EPLBD. Studies have demonstrated the benefits of combining these methods. Ishii et al. found that ESBD resulted in shorter procedure times and a lower frequency of bleeding than EST alone [11]. Additionally, ESBD and ESLBD have been shown to enhance the efficiency of bile duct stone removal. Ding et al. proposed the concept of the “stone extraction tunnel” (SET), which is divided into two segments based on anatomical structure [19]. The proximal segment comprises the distal end of the CBD and the Oddi sphincter within the duodenal wall, whereas the distal segment includes the Oddi sphincter within the papilla of Vater and the papillary opening. Balloon dilatation procedures (EPBD and EPLBD) primarily target the proximal segment, whereas EST mainly targets the distal segment. Therefore, combining these methods allows for complete dilation of the SET, facilitating efficient stone removal. The ability to achieve complete dilation of proximal and distal segments in a short time without replacing the catheter is probably the greatest advantage of this integrated device.

Additionally, the integrated device appears to offer significant benefits for patients with acute cholangitis due to choledocholithiasis. Shortening the procedure time and achieving stone removal within a single session without increasing complications are particularly important for patients with cholangitis. The Tokyo Guidelines 2018 recommend considering single-session stone removal in patients with mild to moderate cholangitis [20]. The results of the present study also showed a significantly higher rate of single-session stone removal and fewer required procedures in the integrated device group, despite a higher proportion of acute cholangitis cases. These results underline the advantage conferred by the integrated device in patients with acute cholangitis due to choledocholithiasis.

Previous studies have established the safety of ESBD and ESLBD [11,21]. The complementary effects of EST and EPBD/EPLBD may further enhance safety. EST reduces the risk of pancreatitis, while the tamponade effect of EPBD/EPLBD minimizes bleeding. This study demonstrated the overall safety of both the conventional and integrated device groups, with no severe adverse events observed. Although there were no significant differences in the overall or individual adverse event rates between the two groups, the integrated device group had no cases of PEP and only mild bleeding cases. PEP is primarily caused by pancreatic juice flow obstruction due to papillary edema or submucosal hemorrhage following ERCP [22]. Previous studies have identified EPBD alone and device insertions under inadequate papillary dilation as risk factors for PEP [23]. The integrated device may have prevented PEP by ensuring adequate dilation of the papillary orifice through EST, reducing device exchanges, and shortening ampullary intervention time, thereby minimizing mechanical stress on the papilla. Bleeding is a major complication of EST, and it can be severe in some cases [4]. Previous studies have shown that ESBD results in less bleeding compared to EST alone, likely due to the incorporation of balloon dilation, which eliminates the need for excessive EST incision [11]. Given the increasing aging population and growing number of patients on antithrombotic therapy [24], devices that reduce bleeding risk are expected to remain valuable in clinical practice.

Some limitations of this study should be considered while interpreting the results. First, the single-center retrospective design conducted at a university hospital may introduce unintended selection, institutional, and operator biases, limiting the generalizability of the findings. Furthermore, propensity score matching could not be performed due to the limited sample size. However, the integrated device group contained more number of patients with poor performance status and periampullary diverticulum, factors associated with procedural difficulty, than the conventional group. Nevertheless, the integrated device group achieved shorter procedure times than the conventional group while maintaining procedural safety. Second, the timing of device use introduced a potential bias. The integrated device group included a relatively high number of patients recently diagnosed with choledocholithiasis. Therefore, the observed shorter procedure times can be attributable to increased endoscopists’ proficiency rather than the device itself. Although not statistically significant, selective biliary cannulation was faster in the integrated device group than in the conventional group. Additionally, we cannot rule out the possibility that the increase in endoscopists’ proficiency over time contributed to the reduction in the use of mechanical lithotripters in the integrated device group. Third, this study could not evaluate the total medical costs. Therefore, the economic efficiency of using this integrated device remains unclear, highlighting the need for further investigations into the associated medical costs.

## 5. Conclusions

Overall complete stone removal rates and safety were comparable in the conventional group and integrated device group. However, the novel integrated device may enhance the efficiency of CBD stone removal using ESBD and ESLBD because eliminates the need to prepare and exchange separate devices.

## Figures and Tables

**Table 1:** Characteristics of the study population.

	Conventional Group (*n* = 106)	Integrated Device Group (*n* = 54)	*p*-Value
Age, median (range), years	81 (27–95)	81 (45–98)	0.51
Sex, male/female, n	55:51	33:21	0.32
Performance status score of 0–1, n (%)	103 (97.2%)	47 (87.0%)	0.03
Antiplatelet or anticoagulant therapy, n (%)	29 (27.4%)	20 (37.0%)	0.28
Cardiovascular disease, n (%)	35 (33.0%)	24 (44.4%)	0.17
History of cerebrovascular disease, n (%)	15 (14.2%)	4 (7.4%)	0.30
Dementia, n (%)	4 (3.8%)	4 (7.4%)	0.44
History of malignant neoplasm, n (%)	27 (25.5%)	11 (20.4%)	0.56
Billroth I reconstruction, n (%)	4 (3.8%)	5 (9.3%)	0.17
Previous history of EST/EPBD/EPLBD	15 (14.2%)	3 (5.6%)	0.12
Periampullary diverticulum, n (%)	38 (35.8%)	30 (55.6%)	0.02
Complications of acute cholangitis, n (%)	67 (63.2%)	42 (77.8%)	0.07
Severity of cholangitis, mild/moderate to severe, n	27/40	20/22	0.55
Complication of gallstone pancreatitis, n (%)	2 (1.9%)	1 (1.9%)	1.00
Number of stones, median (range), n	2 (1–10)	2 (1–10)	0.33
Diameter of stones, median (range), mm	11 (2–24)	10 (2–32)	0.66
Diameter of common bile duct, median (range), mm	12 (5–25)	11 (6–24)	0.12

EST, endoscopic sphincterotomy; EPBD, endoscopic papillary balloon dilation; EPLBD, endoscopic papillary large balloon dilation.

**Table 2:** Summary of procedural details in the two groups.

	Conventional Group *n* = 106	Integrated Device Group *n* = 54	*p*-Value
ESBD (balloon size < 12 mm): ESLBD (balloon size ≥ 12 mm), n	58:48	32:22	0.62
Complete stone removal, n (%)	105 (99.1%)	54 (100%)	1.00
Single-stage stone removal, n (%)	43 (40.6%)	33 (61.1%)	0.02
Total number of sessions for complete stone removal, median (range), n	1 (1–3)	1 (1–3)	0.01
Use of mechanical lithotripsy for stone removal	53 (50.0%)	12 (22.2%)	<0.01
Total procedure time, median (range), minutes	40 (10–119)	27 (12–67)	0.01
Time required for selective biliary cannulation, median (range), minutes	4 (1–87)	3 (1–31)	0.06
Rectal diclofenac administration before procedure	0 (0.0%)	0 (0.0%)	1.00
Pancreatic guidewire method for selective biliary cannulation	16 (15.1%)	6 (11.1%)	0.63
Pancreatic stent placement	4 (3.8%)	0 (0.0%)	0.31
Pre-cutting for selective biliary cannulation	2 (1.9%)	0 (0.0%)	0.55
Length of hospital stay, median (range), days	12 (3–58)	8 (2–30)	0.02

ESBD, endoscopic papillary balloon dilation with endoscopic sphincterotomy; ESLBD, endoscopic papillary large balloon dilation with endoscopic sphincterotomy.

**Table 3:** Incidence of adverse events.

	Conventional Group (*n* = 106)	Integrated Device Group (*n* = 54)	*p*-Value
Adverse events, n (%)	13 (12.3%)	7 (13.0%)	1.00
Pancreatitis, n (%)	3 (2.8%)	0 (0%)	0.55
Bleeding, n (%)	6 (5.7%)	3 (5.6%)	1.00
Perforation, n (%)	0 (0%)	0 (0%)	1.00
Bile leakage, n (%)	0 (0%)	0 (0%)	1.00
Cholecystitis, n (%)	1 (0.9%)	0 (0%)	1.00
Cholangitis, n (%)	0 (0%)	2 (3.7%)	0.11
Deterioration of respiratory condition, n (%)	2 (1.9%)	2 (3.7%)	0.60
Aspiration pneumonia, n (%)	2 (1.9%)	0 (0%)	0.55

**Table 4:** Management of bleeding.

	Immediate Bleeding		Delayed Bleeding	
	Conventional Group	Integrated Device Group	Conventional Group	Integrated Device Group
***Patients, n***	4	2	2	1
Mild/moderate/severe (lexicon criteria)	4/0/0	2/0/0	1/1/0	1/0/0
Blood transfusion	0	0	1	0
***Endoscopic treatment for bleeding, n***				
Diluted epinephrine spray	3	2	1	0
Balloon tamponade	4	0	1	1
HSE injection	0	0	1	0
Clipping	0	0	1	0

HSE, hypertonic saline epinephrine solution.

**Table 5:** Comparison of procedural details and adverse events between the small-stone and large-stone groups.

	Small-Stone Group (*n* = 80)	Large-Stone Group (*n* = 80)	*p*-Value
Complete stone removal, n (%)	80 (100%)	79 (98.8%)	1.00
Single-stage stone removal, n (%)	41 (51.2%)	35 (43.8%)	0.43
Total number of sessions for complete stone removal, median (range), n	1 (1–3)	1 (1–3)	0.27
ESBD (balloon size < 12 mm): ESLBD (balloon size ≥ 12 mm), n	64:16	26:54	<0.01
Conventional device: Integrated device	51:29	55:25	0.62
Use of mechanical lithotripsy for stone removal	20 (25.0%)	45 (56.2%)	<0.01
Total procedure time, median (range), min	28.5 (6–88)	39.5 (10–119)	<0.01
Time required for selective biliary cannulation, median (range), min	4.5 (1–40)	5.0 (1–87)	0.80
Rectal diclofenac administration before procedure	0 (0%)	0 (0%)	1.00
Pancreatic guidewire method for selective biliary cannulation	9 (11.2%)	13 (16.2%)	0.49
Pancreatic stent placement	1 (1.2%)	3 (3.8%)	0.62
Pre-cutting for selective biliary cannulation	1 (1.2%)	1 (1.2%)	1.00
Length of hospital stay, median (range), days	10 (2–32)	11 (4–58)	0.33
Adverse events, n (%)	9 (11.2%)	11 (13.8%)	0.81
Pancreatitis, n (%)	1 (1.2%)	2 (2.5%)	1.00
Bleeding, n (%)	5 (6.2%)	4 (5.0%)	1.00
Perforation, n (%)	0 (0%)	0 (0%)	1.00
Bile leakage, n (%)	0 (0%)	0 (0%)	1.00
Cholecystitis, n (%)	1 (1.2%)	0 (0%)	1.00
Cholangitis, n (%)	2 (2.5%)	0 (3.7%)	0.50
Deterioration of respiratory condition, n (%)	1 (1.2%)	3 (3.8%)	0.62
Aspiration pneumonia, n (%)	0 (0%)	2 (2.5%)	0.50

Small-stone group; stone size ≤ 10 mm. Large-stone group; stone size > 10 mm.

## Data Availability

The datasets used and/or analyzed during the current study are available from the corresponding author (K.K.) on reasonable request.

## Supplementary Materials

The following supporting information can be downloaded at: https://www.mdpi.com/article/10.3390/jcm14175930/s1, Video S1 Common bile duct (CBD) stone removal via endoscopic papillary balloon dilation with endoscopic sphincterotomy. Video S2 Common bile duct (CBD) stone removal via endoscopic papillary large balloon dilation with endoscopic sphincterotomy.

## Abbreviations

The following abbreviations are used in this manuscript:

CBD	Common bile duct
CBDS	Common bile duct stones
EML	Endoscopic mechanical lithotripsy
EPBD	Endoscopic papillary balloon dilation
EPD	Endoscopic papillary dilation
EPLBD	Endoscopic papillary large balloon dilation
ESBD	Endoscopic papillary balloon dilation
ESLBD	Endoscopic papillary large balloon dilation
ERCP	Endoscopic retrograde cholangiopancreatography
EST	Endoscopic sphincterotomy
HSE	Hypertonic saline epinephrine solution
PEP	Post-ERCP pancreatitis
